# Isolation of an Orally Active Insecticidal Toxin from the Venom of an Australian Tarantula

**DOI:** 10.1371/journal.pone.0073136

**Published:** 2013-09-11

**Authors:** Margaret C. Hardy, Norelle L. Daly, Mehdi Mobli, Rodrigo A. V. Morales, Glenn F. King

**Affiliations:** Institute for Molecular Bioscience, University of Queensland, St Lucia, Australia; Universidad de Granada, Spain

## Abstract

Many insect pests have developed resistance to existing chemical insecticides and consequently there is much interest in the development of new insecticidal compounds with novel modes of action. Although spiders have deployed insecticidal toxins in their venoms for over 250 million years, there is no evolutionary selection pressure on these toxins to possess oral activity since they are injected into prey and predators via a hypodermic needle-like fang. Thus, it has been assumed that spider-venom peptides are not orally active and are therefore unlikely to be useful insecticides. Contrary to this dogma, we show that it is possible to isolate spider-venom peptides with high levels of oral insecticidal activity by directly screening for *per os* toxicity. Using this approach, we isolated a 34-residue orally active insecticidal peptide (OAIP-1) from venom of the Australian tarantula *Selenotypus plumipes*. The oral LD_50_ for OAIP-1 in the agronomically important cotton bollworm *Helicoverpa armigera* was 104.2±0.6 pmol/g, which is the highest *per os* activity reported to date for an insecticidal venom peptide. OAIP-1 is equipotent with synthetic pyrethroids and it acts synergistically with neonicotinoid insecticides. The three-dimensional structure of OAIP-1 determined using NMR spectroscopy revealed that the three disulfide bonds form an inhibitor cystine knot motif; this structural motif provides the peptide with a high level of biological stability that probably contributes to its oral activity. OAIP-1 is likely to be synergized by the gut-lytic activity of the *Bacillus thuringiensis* Cry toxin (*Bt*) expressed in insect-resistant transgenic crops, and consequently it might be a good candidate for trait stacking with *Bt*.

## Introduction

Despite intensive control measures, insect pests reduce world crop yields by 10–14% annually [1], damage 9–20% of stored products [2], and vector a wide variety of diseases of human and veterinary importance [3]. Despite the introduction of biological control methods such as transgenic crops, chemical insecticides remain the dominant method of controlling these insect pests. Every dollar invested in chemical insecticides returns approximately $4 in protection from crop pests, representing an annual saving of ∼$40 billion annually in the United States alone [4].

Contrary to the one-dimensional view of pesticides as broad-spectrum and persistent, recently developed insecticides are highly selective for insect pests [5]. However, because extant chemical insecticides act on a very small number of molecular targets, more than 500 species of arthropods, including most key disease vectors, have become resistant to one or more classes of insecticide [6]. The widespread development of insecticide resistance, together with the de-registration of key insecticides due to perceived ecological and human health risks [7], has created an urgent demand for new insecticidal compounds with novel mechanisms of action.

Over the past decade, there has been increasing interest in the potential of insecticidal proteins as bioinsecticides because of their potentially high phyletic selectivity, low production cost, and the possibility of incorporating transgenes encoding these proteins into plants [8], [9] and entomopathogens [10]. In particular, Cry proteins (δ-endotoxins) isolated from the bacterium *Bacillus thuriengiensis* have had a major worldwide impact on insecticide use. Cry proteins act by forming pores in the insect midgut membrane that eventuate in osmotic shock and cell death [11]. Transgenes encoding Cry proteins have been incorporated into a variety of crops, including cotton, corn, and potato, and in many cases this has substantially improved yields and reduced chemical insecticide use [12]. However, the Cry proteins used in transgenic plants have a relatively narrow host range, being primarily useful against lepidopteran pests, and resistance has been reported in some key pest species [13], [14]. Thus, there is significant interest in the isolation of novel insecticidal proteins with unique modes of action and wider phyletic selectivity.

Spider venoms are arguably the greatest natural reservoir of insecticidal toxins. Spiders are the most speciose venomous animal and, along with predatory beetles, they are the most abundant terrestrial predators [15]. Individual spider venoms can contain more than a thousand peptide toxins [16], and most of these are likely to have insecticidal activity. Indeed, numerous insecticidal peptide toxins have been isolated from spider venoms with activity against a wide range of insect orders [6], [15], [17]. However, since spiders inject their venoms into prey using a hypodermic needle-like fang, there is no evolutionary selection pressure on these toxins to possess oral activity. Very few insecticidal toxins from spider venom have been tested for *per os* activity, which has led to general acceptance of the dogma that they are unlikely to be orally active. However, it has been demonstrated that at least some spider-venom peptides can be orally active [18], which encouraged us in the present study to develop a direct screen for isolating orally active insecticidal peptide toxins from spider venom.

We recently showed that the venom of the Australian tarantula *Selenotypus plumipes* Pocock (Araneae: Theraphosidae) is potently insecticidal [19]. By screening this venom for *per os* activity, we isolated an orally active insecticidal peptide (OAIP-1) that is highly lethal to termites, mealworms, and the cotton bollworm. On a molar basis, OAIP-1 is equipotent with synthetic pyrethroids and it acts synergistically with neonicotinoid insecticides. The 3D structure of this 34-residue peptide, which we determined using NMR spectroscopy, revealed the presence of a cystine knot motif that typically confers extreme chemical and thermal stability as well as resistance to proteases [20]. Consistent with this finding, we show that OAIP-1 remains completely intact for at least one week at temperatures up to 30°C and is stable for hours in insect hemolymph.

The current study indicates that it is possible to isolate insecticidal peptides with high levels of oral activity from the venom of spiders and most likely other venomous animals that prey on insects (e.g., centipedes and scorpions). These orally active peptides might have potential as standalone bioinsecticides or alternatively transgenes encoding the peptides could be used to engineer insect-resistant transgenic plants or enhance the efficacy of entomopathogens.

## Materials and Methods

### Venom fractionation and peptide sequencing

Venom was collected from *S. plumipes* spiders and lyophilized as previously described [19]. Toxins were isolated by fractionating 500 µL of a 10-fold dilution of the crude venom using a Vydac C_18_ analytical reverse-phase high pressure liquid chromatography (RP-HPLC) column (5 µm, 4.6×250 mm; Grace Davison, Deerfield, IL). Solvent A was 0.1% trifluoroacetic acid (TFA) in water and Solvent B was 0.1% TFA in acetonitrile. Toxins were eluted at a flow rate of 1.0 mL/min using a linear gradient of 5% Solvent B for 5 min, 5–25% Solvent B over 20 min, then 25–50% Solvent B over 48 min. Individual fractions were lyophilized, resuspended in 100 µL of water, and further purified using cation exchange chromatography on a MonoS HR5/5 column (50×100 mm; GE Healthcare, Piscataway, NJ). Buffer A was 0.1 M NaCl (pH 5.5) and Buffer B was 2 M NaCl (pH 5.5); the gradient used was 5% Buffer B for 15 min followed by 5–45% Buffer B over 40 min. Toxins were desalted using RP-HPLC then lyophilized and stored at −20°C.

Mass spectrometry was performed on an Applied Biosystems 4700 MALDI TOF-TOF Proteomics Analyzer (Carlsbad, CA) using 2 µL of an RP-HPLC fraction and 0.8 µL of 10 mg/mL α-cyano-4-hydroxycinnamic acid (CHCA) matrix (dissolved in 50% acetonitrile/50% water/0.1% TFA) to verify peptide masses.

Individual toxins were reduced and alkylated with 4-vinylpyridine (4VP) using a modified protocol [21]. Purified toxins (20–30 µg) were dissolved in 100 µL of Milli-Q water then an equal volume of 4VP buffer (0.25 M Tris, 2 mM EDTA, 10 mM dithiothreitol (DTT), pH 8.0) was added. The solution was incubated at 65°C for 20 min to reduce all disulfide bonds. After 20 min, 5 µL 4VP and 20 µL acetonitrile were added. The alkylation reaction was allowed to proceed in the dark at ambient temperature for 60 min. Alkylated toxins (45–450 pmol per sample) were sent to the Australian Proteome Analysis Facility (APAF, Sydney, NSW, Australia) and the Adelaide Proteomics Centre (APC, Adelaide, Victoria, Australia) for N-terminal sequencing.

### Determination of insecticidal activity

In order to determine which venom fractions were orally active in termites, lyophilized RP-HPLC fractions were fed to termites (*Coptotermes acinaciformis* (Froggatt), Isoptera: Rhinotermitidae) collected from colonies maintained by the Department of Primary Industries and Fisheries (Long Pocket, Indooroopilly, QLD, Australia). Termites were fed a 20% α-cellulose matrix (Sigma-Aldrich, St. Louis, MO) mixed with water; the toxin was dissolved in water, and 20 µL was added to the cellulose matrix to a final concentration of 350 nmol/g, and then pipetted into Petri dishes. After the cellulose matrix had dried (to prevent termites from drowning in wet bait), nine worker termites and one soldier termite were added to each dish; each toxin dose was replicated three times. As a comparison, toxins were also injected into mealworms at a concentration of 350 nmol/g in three replicates of 10 insects.

Mealworms were purchased from Pisces Enterprises (Kenmore, QLD, Australia). Insects between 3^rd^ and 4^th^ instar (∼180 mg/individual) were used. For each mealworm, 2.6 µL of toxin diluted in ultrapure water was injected into the metathoracic pleurite. Injections were performed using a 29.5 gauge insulin syringe (B–D Ultra-Fine, Terumo Medical Corporation, Elkton, MD). Three replicates of 10 insects were used for each toxin concentration, and the same number of control insects were injected with ultrapure water and maintained under the same conditions.

Chicken feed was provided *ad libitum* for mealworm food. A Whatman filter paper saturated with distilled water was used to maintain humidity in all mealworm experiments except feeding assays, which used the synthetic orally active insecticidal peptide 1 (sOAIP-1) in the agar diet described below. The agar diet allowed a homogenous mixture of toxin and diet to be prepared so preferential feeding on untreated diet would not be possible [22]. All insects were maintained at 24.5°C in the dark at ambient humidity in sterile Petri dishes.

For feeding assays, an agar-based insect diet was created based on literature information (Table S1). Instead of using a separate preparation as a vitamin supplement, the commercially available children's vitamin Pentavite (Bayer, Leverkusen, Germany) was added after the agar had cooled below 65°C. Mealworms were fed 100 µL of the agar diet, with or without 20 µL toxin (or water for untreated controls). For the choice test, the same size Petri dish was divided in half and 50 µL of either toxin-treated or untreated agar was pipetted onto each half and mortality was recorded after 48 h. Cotton bollworms (*Helicoverpa armigera*, Lepidoptera: Noctuidae) were fed 5 µL toxin (or water for untreated controls) in 20 µL of agar diet, and maintained in 12-well tissue culture dishes. For assays of synergism using 100 pmol imidacloprid (Sigma-Aldrich) and 100 pmol sOAIP-1, 20 µL of one toxin or 10 µL of each toxin was incorporated into the agar diet for *H. armigera*. After all the treated diet was consumed, untreated diet was supplied *ad libitum*.

In all experiments, the mortality of untreated insects was used to correct the data for mortality due to injection of toxin or incorporation in the diet. The correction was made using Abbott's formula [23], Corrected% Mortality  = (1−*T*_n_/*T*_C_)×100, where *T*_n_ is the percent mortality in the treated group and T_C_ is the percent mortality in the untreated control group.

Mealworms injected with sOAIP-1 were observed at 5, 30, and 60 min intervals to record behavioral changes. A numeric score was assigned to each state and averaged to provide an indication of the effect of the toxin (adjusted for the effects of the injection via the insects injected with water). The criteria used to score the response of injected insects to the toxin are summarized in Table S2.

### Transcriptome assembly

Four venom glands from two *S. plumipes* spiders were prepared and total RNA was immediately extracted using TRIzol (Invitrogen, Carlsbad, CA). The concentration of total RNA was measured using a Nanodrop (ND-1000, ThermoScientific, Wilmington, DE) and the quality confirmed using a Bioanalyzer 2100 (Agilent Technologies, Santa Clara, CA). An Oligotex Direct mRNA Mini Kit (Qiagen, Hilden, Germany) was used to isolate poly A^+^ mRNA from the total RNA. Elution was performed first in 5 mM Tris-HCl (pH 7.5), and subsequently samples were precipitated with RNAse-free glycogen, sodium acetate, and ethanol. Samples were resuspended again in RNAse-free water, and the RNA concentration and quality were measured using the Nanodrop and Bioanalyzer. The mRNA (227 ng) was submitted for pyrosequencing using the Roche 454 GS-FLX platform (Roche, Basel, Switzerland) at the Australian Genome Research Facility (Brisbane Node, The University of Queensland, St. Lucia, QLD, Australia).

Raw 454 reads were assembled using SeqMan NGen (v2, DNAStar, Madison, WI). After assembly, the sequences obtained from N-terminal sequencing of OAIP-1 were BLASTed against the raw 454 data. Sequence hits were then matched to contigs assembled using SeqMan NGen. The complete OAIP-1 transcript, which included the signal sequence and propeptide region, was then isolated from the assembled data using *Geneious* software, v. 5.1.

### Solid-phase peptide synthesis

Synthetic OAIP-1 (sOAIP-1) was produced via Fmoc solid-phase peptide synthesis. Fmoc–protected L-amino acids Arg(Tos), Asn(Trt), Asp(OtBu), Ala, Cys(Trt), Gln(Trt), Glu, Gly, His(Trt), Ile, Leu, Lys(Boc), Met, Phe, Pro, Ser(tBu), Thr, Tyr(tBu), Trp and Val were purchased from Novabiochem (Merck, Darmstadt, Germany). Amino acid-loaded Fmoc Wang resins were obtained from the Peptide Institute (Osaka, Japan). *N,N*-dimethylformamide (DMF), TFA, *N,N*-diisopropylethylamine (DIEA) and piperidine were obtained from Auspep (Tullamarine, VIC, Australia). Triisopropylsilane (TIPS) and diethylether (Sigma-Aldrich), and acetonitrile (Merck) were obtained from commercial suppliers.

sOAIP-1 was synthesized on Wang polystyrene resin preloaded with the first C-terminal amino acid residue (0.2 mmol/g). Chain assembly was performed following a previously established *in situ* neutralization protocol [24]. The process was carried out using a Symphony Automatic Peptide Synthesizer (Protein Technologies, Inc., Tucson, AZ). sOAIP-1 was then de-protected and cleaved from the solid resin with a solution of TFA∶TIPS∶water at 90∶5∶5 ratio for 3 h and evaporated under a stream of N_2_. The desired product was precipitated in cold diethylether and filtered. The retained crude peptide product was dissolved in an aqueous acetonitrile solution (50% acetonitrile, 0.1% TFA). Crude peptide solutions were lyophilized.

sOAIP-1 was then purified via RP-HPLC using a linear acetonitrile gradient (15–40% Solvent B over 25 min); the toxin eluted at ∼28% Solvent B. Mass spectrometry was performed as described above to confirm that a peptide of the correct mass had been produced. The toxin (0.1 mg/mL) was folded overnight at room temperature in an ammonium bicarbonate redox buffer (0.1 M NH_4_HCO_3_, pH 8.0, 5 mM reduced glutathione, 0.5 mM oxidized glutathione). A linear acetonitrile gradient (15–30% over 40 min) was used in a final RP-HPLC step to purify the folded peptide to >98% homogeneity.

### Structure determination

Lyophilized sOAIP-1 was resuspended in phosphate buffer (10 mM H_2_KPO_4_, pH 5.8 in either 95% H_2_O:5% D_2_O or 100% D_2_O) at a final concentration of 700 µM. Samples (300 µL) were filtered using a 0.22 µM Ultrafree-MC centrifugal filter (Millipore, Billerica, MA) and added to a susceptibility-matched 5 mm outer-diameter microtube (Shigemi, Osaka, Japan). A high-resolution 1D NMR spectrum and 2D ^1^H-^1^H TOCSY, ^1^H-^1^H NOESY, ^1^H-^1^H DQF-COSY, ^1^H-^15^N HSQC, and ^1^H-^13^C HSQC spectra were acquired at 298 K using a 900 MHz AVANCE NMR spectrometer (Bruker, Karlsruhe, Germany) equipped with a cryogenically cooled probe. All spectra were recorded with an interscan delay of 1 s. NOESY spectra were acquired with mixing times of either 200 ms (D_2_O sample) or 130 ms (H_2_O sample). TOCSY spectra were acquired with isotropic mixing periods of either 90 ms (H_2_O) or 70 ms (D_2_O). Standard Bruker pulse sequences were used with a WATERGATE pulse sequence for solvent suppression. NMR data were processed using nmrPipe and the Rowland NMR Toolkit.

TALOS+ was used to predict protein backbone torsion angles from the NMR chemical shifts [25], [26]. The 2D NOESY spectrum was automatically assigned and an ensemble of structures calculated without manual intervention using the program CYANA [27]. Torsion-angle restraints from TALOS+ were used in the structure calculations. The disulfide bond connectivities were unambiguously determined to be Cys2–20, Cys9–25, and Cys19–30 based on preliminary structure calculations. Distance restraints for the disulfide bonds were used in subsequent rounds of structure calculation as described previously [28]. PROCHECK was used to analyze the stereochemical quality of the final structures [29], which were visualized using PyMol software (http://www.pymol.org).

### In vitro stability assessment

Fourth-instar *H. armigera* larvae were decapitated and the gut was removed using forceps. The carcasses were spun in a benchtop centrifuge (14,000 *g* for 10 min) to separate the hemolymph from exoskeleton. For each time point, 200 µL of undiluted hemolymph was mixed with 30 µg of sOAIP-1. The hemolymph/sOAIP-1 solution was maintained in the dark at room temperature, and immediately before RP-HPLC analysis the hemolymph/sOAIP-1 solution was again spun in a benchtop centrifuge (14,000 *g* for 10 min). After centrifugation, 30 µg of a control peptide, ω-HXTX-Hv1a [30], was added to aid quantification and the sample was filtered using a 0.45 µM filter. The toxins were separated by RP-HPLC using a linear acetonitrile gradient (5–40% over 40 min), and the identity of each toxin was confirmed using mass spectrometry. The percentage of intact sOAIP-1 present at each time point was determined by comparing the area of the sOAIP-1 peak to that at zero time, both measured relative to the area of the ω-HXTX-Hv1a peak.

### Thermal and chemical stability of OAIP-1

The stability of OAIP-1 was determined over a range of temperatures and pH conditions. The pH range was 3–8, and samples were prepared by adding 100 ng of OAIP-1 dissolved in water to an equivalent volume of pH buffer. The pH buffers comprised 20 mM sodium citrate (pH 3 and 4), 20 mM sodium acetate (pH 5), 20 mM 2-ethanesulfonic acid (MES, pH 6), 20 mM sodium phosphate (pH 7), or 20 mM Tris (pH 8). pH stability was examined over seven days at ambient temperature (22°C). The temperature range examined was −20 to 50°C; samples were dissolved in water and held in the dark in a monitored freezer (−20°C), at ambient temperature (22°C), in a temperature-controlled incubator (30 and 37°C), or on a hotplate (50°C). Each pH and temperature condition was sampled at 0, 1, 2, 5, 24, 48, 72, 120, and 168 h, (*n* = 2).

pH samples were analyzed by LC-MS using a Nexera UHPLC system (Shimadzu, Japan) coupled to a TripleTOF 5600 mass spectrometer (AB SCIEX, USA). Samples (12 µl) were injected onto a Zorbax C18 column (2.1 mm×100 mm, particle size 1.8 µm; Agilent Technologies, USA) and peptides were eluted at a flow rate of 250 µL/min using a linear gradient of 5–40% Solvent B over 10 min. Solvent A was 0.1% formic acid while Solvent B was 90% water/10% acetonitrile/0.1% formic acid. Mass spectral data were acquired over the m/z range 850–1350 and processed using Analyst TF 1.6 software (AB SCIEX). Intact OAIP-1 was identified by its UHPLC retention time (4.6 min) and from the major isotope peaks of the +4 protonated species (m/z 930.36–930.60).

Samples from the temperature stability experiments were analyzed via MALDI TOF-TOF mass spectrometry using a 4700 Proteomics Analyzer (Applied Biosystems, USA) in order to identify intact OAIP-1. Samples (2 µL) were mixed with 0.8 µL of α-cyano-4-hydroxycinnamic acid (CHCA) matrix (10 mg/mL dissolved in 50% acetonitrile/50% water/0.1% TFA) for mass spectral analysis.

## Results

### Identification and purification of active toxins

**Figure 1:**
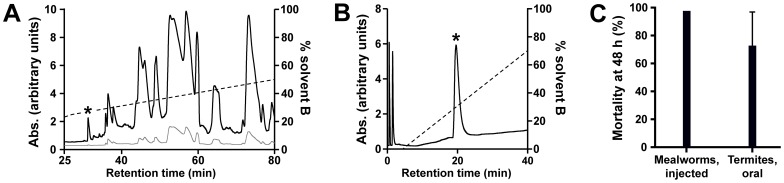
Isolation of an orally active insect toxin from spider venom. (A) RP-HPLC chromatogram showing fractionation of crude venom from the Australian tarantula *Selentypus plumipes*. An asterisk highlights the fraction that displayed oral termiticidal activity. (B) Chromatogram from cation exchange fractionation of the active RP-HPLC fraction shown in (A). An asterisk highlights the fraction with oral termiticidal activity. (C). Insecticidal assay of native OAIP-1. The peptide was injected into larvae of the mealworm beetle (*Tenebrio molitor*) at a dose of 3 pmol/g or fed to termites (*Coptotermes acinaciformis*) at a dose of 350 nmol/g. Each column represents the mean ±SD of three replicates of 10 insects.

Fractionation of *S. plumipes* venom using RP-HPLC yielded ∼50 peaks that eluted before 60% Solvent B. The RP-HPLC fraction marked with an asterisk (*) in Fig. 1A was shown to have activity when fed to termites, indicating that this fraction must contain orally-active insecticidal components.

The RP-HPLC fraction with oral activity was further fractionated using an orthogonal cation exchange chromatography step (Fig. 1B) in order to isolate the active peptide, which was then desalted using RP-HPLC. The active peptide was given the trivial name orally active insecticidal peptide 1 (OAIP-1); its rational name based on the nomenclature for spider toxins [31] that is used in both UniProt [32] and ArachnoServer [33] is U_1_-TRTX-Sp1a. After confirming oral activity in termite feeding assays, the active OAIP-1 peptide was reduced and the resulting free cysteines were alkylated using 4VP in order to facilitate N-terminal sequencing by Edman degradation. Since a single vinyl-pyridine moiety is covalently attached to each cysteine residue during this process, the increase in peptide mass following the alkylation procedure provides a measure of the number of cysteines (and hence the number of disulfide bonds) in each OAIP. Based on these peptide-mass analyses, it was determined that OAIP-1 contains six cysteine residues (i.e., three disulfide bonds).

### Preliminary oral insecticidal activity

OAIP-1 was fed to termites (mean individual weight 3.61±0.3 mg) at an approximate dose of 350 nmol/g and injected into mealworms (mean individual weight 244.0±5.0 mg) at an approximate dose of 3 pmol/g. At these doses, the purified OAIP-1 produced mortality above 70% in both insect species (Fig. 1C).

### OAIP sequence determination

**Figure 2:**
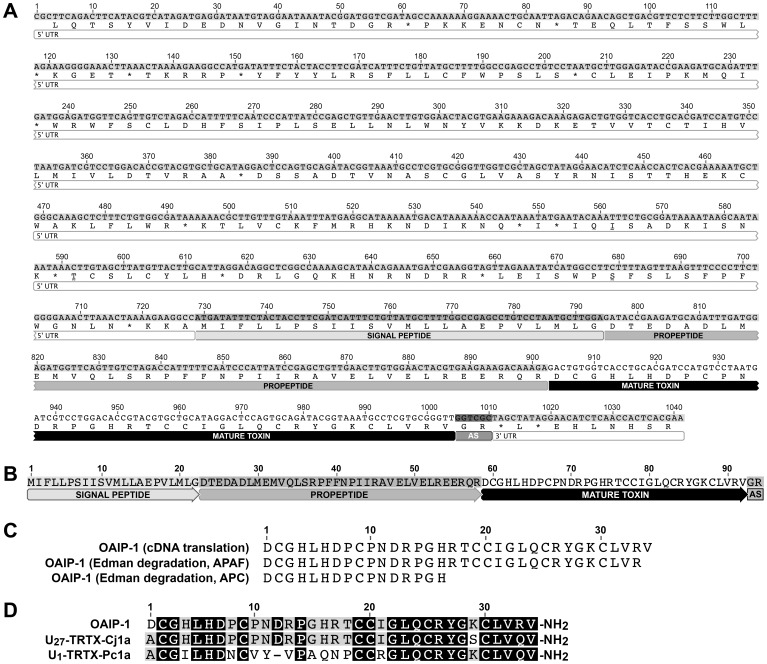
Primary structure of OAIP-1. (A) Sequence of transcript encoding the OAIP-1 prepropeptide precursor isolated from an *S. plumipes* venom-gland cDNA library. The 3′ and 5′ untranslated region (UTR), signal sequence, propeptide region, and mature toxin are labeled. The “GR” dipeptide sequence at the end of the mature toxin sequence is labeled AS (amidation signal) as it is a signal for C-terminal amidation. (B) Amino acid sequence of OAIP-1 prepropeptide precursor obtained from *in silico* translation of the cDNA sequence shown in panel (A). (C) Comparison of the amino acid sequence of the mature OAIP-1 toxin obtained from *in silico* translation of the venom-gland prepropeptide transcript with the N-terminal sequences obtained from Edman degradation of the native toxin at the APAF and APC protein sequencing facilities. (D) Alignment of OAIP-1 primary structure with the two closest hits obtained from a BLAST search against the ArachnoServer database. Identical residues are highlighted by white letters on a black background, while residues that are identical in two of the three sequences are shown on a gray background.

Partial and complete sequences of native OAIP-1 were obtained from samples submitted to APC and APAF, respectively (Fig. 2C). These sequences were BLASTed against the 329,028 raw sequences obtained from a transcriptome prepared from the venom glands of *S. plumipes*. Once the BLAST algorithm identified a match to a raw 454 read, the partial sequence was traced to an assembled contig and the complete sequence of the toxin-encoding transcript was obtained.

Analysis of the OAIP-1 transcript revealed that it is initially produced as a 94-residue prepropeptide that is posttranslationally processed to produce the 34-residue mature toxin (Fig. 2A). The SignalP 4.0 Server [34] was used to predict the signal peptide cleavage site, while the propeptide cleavage site could be determined unequivocally from the N-terminal sequence information obtained for the fully processed toxin. The cDNA sequence of the complete transcript is shown in Fig. 2A, and the translated protein sequence is shown separately in Fig. 2B. The “GR” at the C-terminus of OAIP-1 is a signal for C-terminal amidation, and mass spectrometric analysis of the purified mature toxin is consistent with an amidated C-terminal residue. The OAIP-1 sequence obtained from *in silico* translation of the precursor mRNA is in complete agreement with the N-terminal protein sequence obtained from Edman degradation, as shown in the sequence alignment in Fig. 2C.

### Search for OAIP homologs

ArachnoServer is a manually curated database that provides information on the sequence, structure, and function of all known protein toxins from spiders [33], [35]. A BLAST search of the ArachnoServer database (www.arachnosever.org) using both mature OAIP-1 toxin as well as the complete OAIP-1 transcript revealed two close sequence matches (Fig. 2D). The closest match, with 91% identity, was U_27_-TRTX-Cj1a, a toxin with unknown function and molecular target identified in a cDNA library prepared from the venom glands of the Chinese tarantula *Chilobrachys jingzhao* [36]. The next best match with 62% identity was U_1_-TRTX-Pc1a, a toxin from the Trinidad chevron tarantula *Psalmopoeus cambridgei* that was reported to inhibit intra-erythrocyte development of the malaria parasite *Plasmodium falciparum* [37]. Like OAIP-1, U_1_-TRTX-Pc1a is C-terminally amidated, and the transcript encoding U_27_-TRTX-Cj1a also contains a C-terminal amidation signal. Since all three toxins were isolated from theraphosid spiders (tarantulas), they are likely to be orthologous.

### Insecticidal assays

**Figure 3:**
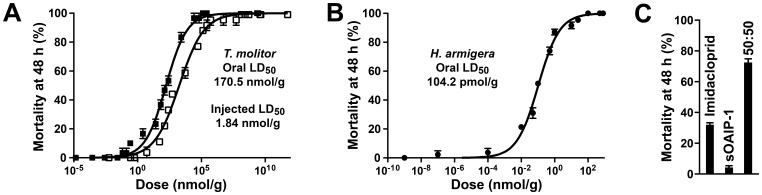
Insecticidal activity of synthetic OAIP-1. (A) Dose-response curves resulting from administration of sOAIP-1 to mealworms (larval *T. molitor*) via injection (▪) or feeding (□). (B) Dose-response curve resulting from feeding sOAIP-1 to cotton bollworms (larval *H. armigera*) (•). The calculated LD_50_ values are shown. (C) Mortality observed at 48 h after feeding 100 pmol imidacloprid, 100 pmol sOAIP-1, or a 50∶50 mixture of these compounds into *H. armigera*. Each data point is the mean ±SEM of three replicates of 10 individuals.

The injected and *per os* activity of sOAIP-1 was initially determined using mealworms because of their previously established sensitivity to spider toxins [19]. Dose-response curves, adjusted for the mortality of untreated controls, were used to calculate LD_50_ values, which were 1.84±0.8 nmol/g for injected toxin and 170.5±0.2 nmol/g for oral administration (i.e., the toxin was ∼90-fold less potent when delivered *per os*) (Fig. 3A). Remarkably, and somewhat surprisingly, sOAIP-1 was much more potent against the cotton bollworm *H. armigera*, a pernicious crop pest, with a *per os* LD_50_ of 104.5 pmol/g (Fig. 3B).

We compared the mortality obtained when *H. armigera* were fed either 100 pmol of the widely used neonicotinoid insecticide imidacloprid (the approximate LD_50_ value calculated for these Lepidoptera at their instar and weight) or 100 pmol sOAIP-1, or a 50% mixture of each (i.e., 50 pmol of each insecticide, Fig. 3D). The 50∶50 mixture yielded mortality higher than either insecticide individually (72±5%, compared to 31±3% for imidacloprid and 4±3% for sOAIP-1). The two insecticides are clearly synergistic, indicating that they likely act on different molecular targets, with the combination exhibiting a greater than two-fold increase in activity over imidacloprid alone.

### Feeding choice test with OAIP-1

**Figure 4:**
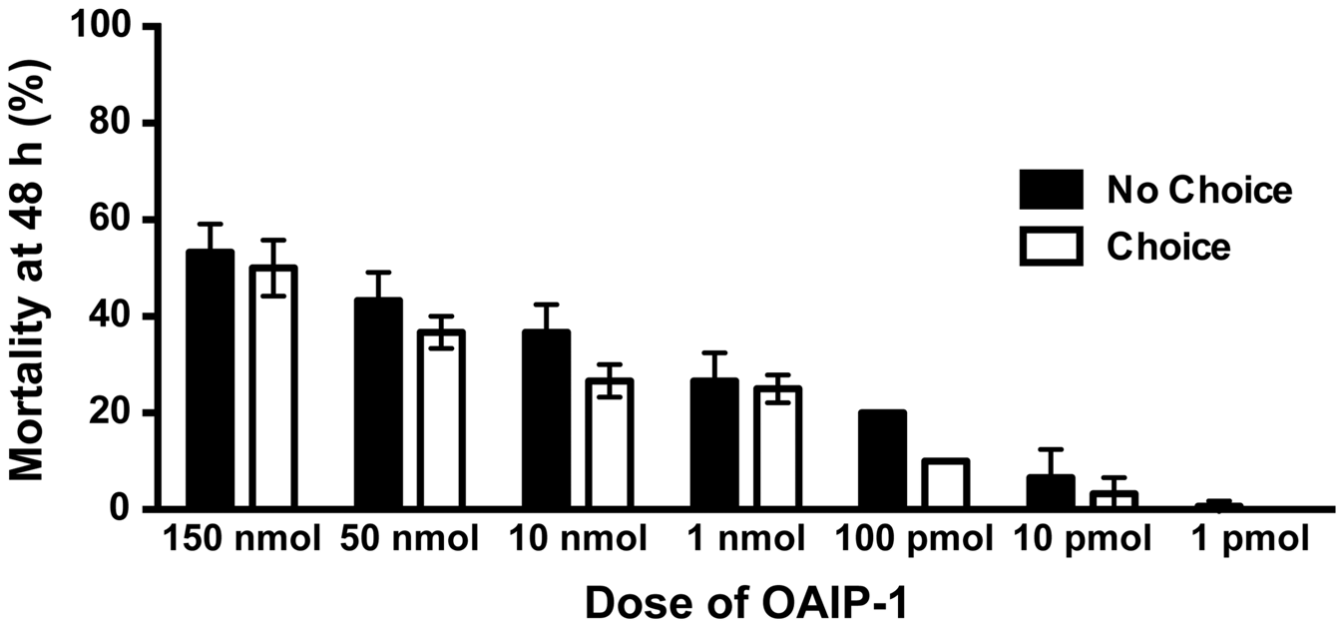
Choice test with OAIP-1. Mortality of *T. molitor* larvae (mealworms) determined at 48 h after insects were simultaneously offered toxin-treated and untreated agar. The toxin concentration in the treated agar ranged from 1 mmol to 1 pmol, and the data represent the mean and SEM of three replicates of 10 individuals for each dose. The data correlate well with the oral toxicity of sOAIP-1 in a non-choice test (Fig. 3A); the mortality at the same dose in the choice test is approximately the same as that observed in the non-choice test. Mortality at all but the lowest two doses (10 and 1 pmol) was significantly greater than the untreated agar control (P<0.01). Columns represent the mean ±SD for three replicates of 10 insects for each dose.

In addition to toxicity assays, a choice test was conducted to determine whether sOAIP-1 is repellent. This involved exposing a group of mealworms to both toxin-treated and untreated agar (Fig. 4); if both agars were fed on equally, it would suggest that sOAIP-1 is not repellent. Conversely, if the toxin-treated agar was preferentially consumed, it might indicate that sOAIP-1 acts as an attractant.

According to Dunnett's Multiple Comparison Test [38], the mortality at 48 h was significantly elevated (P<0.01) above that of the control for all doses except at the lowest two doses (10 and 1 pmol toxin). At 1 pmol toxin, there was no mortality. This indicates that mealworms fed voluntarily on toxin-treated agar, even though untreated agar was available to them. The data concur with what was observed when mealworms were offered only treated agar (Fig. 3A); the mortality observed in the choice test (where 50% untreated and 50% treated agar was offered) was approximately half that seen in the non-choice test (where only toxin-treated agar was available). This suggests that sOAIP-1 is neither a repellent that repels insects nor an attractant that is preferentially consumed by insects.

### Phenotypic response to OAIP-1

**Figure 5:**
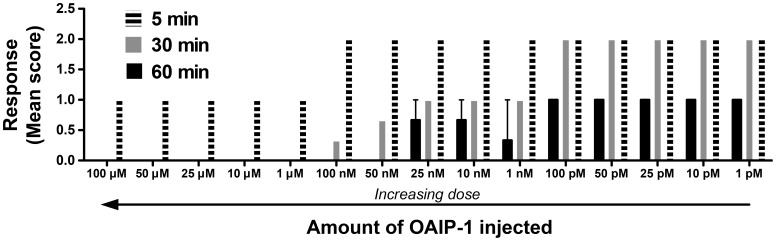
Phenotypic response of insects to OAIP-1. *T. molitor* larvae (mealworms) were monitored 5, 30, and 60 min following injection of sOAIP-1 (horizontally striped, grey, and black bars, respectively). The response was scored relative to the control as excitatory (prolonged muscle spasms), excitation to the point of paralysis (spasms so severe the insect was unable to move independently), or death/moribund (dead or, if alive, the insect was unable to right itself when turned on its back). See Table S2 for details for the scoring matrix. No dose produced a depressed state at any of the time points. Columns represent the mean ±SEM of three replicates of 10 insects for each dose.

A scored response test was used to quantify the phenotypic response to OAIP-1 by comparing the response of the insects injected with toxin to that of insects injected with water. Phenotypic responses were observed in mealworms 5, 30, and 60 min following injection of sOAIP-1 (Fig. 5); these were the same insects used to construct the dose-response curve. A score close to zero represents dead or moribund insects; a score of 2 indicates insects that exhibit an excitatory response but are not paralyzed and can still move independently. Insects scored at 1 exhibited excitatory paralysis, which is categorized as an overstimulation of the nervous system that included constant shaking, rapid leg movements, and uncontrollable spasms resulting in an inability of the insect to move independently (e.g., to right itself when turned upside down).

Many arachnid toxins inhibit presynaptic voltage-gated ion channels [7], and this typically induces a depressant response as synaptic transmission is inhibited [39]; these toxins would receive a negative score in the phenotypic response assay. OAIP-1, with scores of 0.5–2 depending on dose and duration (Fig. 5), clearly does not have this mode of action. Rather, the excitatory phenotype induced by sOAIP-1 suggests that it might be an activator of presynaptic voltage-gated ion channels (e.g., it may be an agonist or a gating modifier that slows down channel inactivation) or an agonist of a postsynaptic receptor (the mode of action of neonicotinoid insecticides such as imidacloprid).

### Stability of OAIP-1

**Figure 6:**
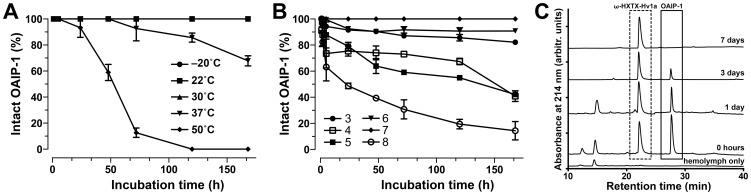
Stability of OAIP-1. (A) Thermal stability of sOAIP-1 over 7 days. Note that the data obtained at −20°C, 22°C, and 30°C overlap completely since OAIP-1 is 100% intact at these temperatures at all time points. OAIP-1 only degrades at temperatures of 37°C and higher. (B) Stability of sOAIP-1 over a range of different pH conditions. The toxin is least stable at alkaline pH. (C) A series of RP-HPLC chromatograms showing fractionation of undiluted hemolymph from *H. armigera* larvae (cotton bollworms) at various times following addition of 30 µg sOAIP-1 (highlighted in the solid box). Immediately before RP-HPLC fractionation, 30 µg of ω-HXTX-Hv1a (dashed box) was added to each sample for the purposes of quantification. In all experiments shown in panels A–C, intact OAIP-1 was identified using mass spectrometry.

OAIP-1 remained completely intact over a period of 7 days at temperatures ranging from −20°C to 30°C (Fig. 6A). Slow degradation occurred after 2 days at 37°C but the peptide was nevertheless 60% intact after 7 days at this temperature (Fig. 6A). Thus, it is likely that OAIP-1 can be stored for long periods of time at temperatures below 37°C. Degradation was rapid at 50°C, a temperature well above the most extreme conditions that OAIP-1would likely experience in the field, with no intact peptide evident after 5 days at this temperature (Fig. 6A).

At 22°C, OAIP-1 was completely stable over 7 days at pH 7 and very little degradation was evident at pH 3 and 6 (Fig. 6B). Surprisingly, the peptide was less stable at the intermediate acidic pH values of 4 and 5, with about 60% degradation over 7 days (Fig. 6B). OAIP-1 was least stable under alkaline conditions, with only ∼15% remaining intact after 7 days at pH 8 (Fig. 6B). This was expected as the p*K*_a_ of free cysteine is ∼8.3 and consequently disulfide-rich peptides generally become more susceptible to disulfide opening and shuffling at pH values approaching or exceeding this value.

We also determined the stability of sOAIP-1 *ex vivo* in insect hemolymph at ambient temperature (23°C), which has more direct relevance to its application as a bioinsecticide. Immediately prior to RP-HPLC analysis of each hemolymph sample, 30 µg of the 37-residue insecticidal peptide ω-HXTX-Hv1a was added to aid quantitation of the sOAIP-1 level. RP-HPLC analysis of the hemolymph samples (Fig. 6C) revealed that approximately 40% of sOAIP-1 remained intact after 24 h exposure to hemolymph proteases, while 90% of the peptide was degraded after 72 h and none remained intact after a one-week incubation in undiluted hemolymph (Fig. 6C).

### Determination of the 3D structure of OAIP-1

NMR spectroscopy was used to determine the 3D structure of sOAIP-1. 2D homonuclear TOCSY, NOESY, and COSY spectra as well as natural abundance ^1^H-^15^N and ^1^H-^13^C HSQC spectra were acquired at 298 K using a 900 MHz Bruker spectrometer. Sequence-specific resonance assignments were made using TOCSY and NOESY spectra; the natural abundance HSQC spectra were primarily used to obtain ^15^N, ^13^Cα, and ^13^Cβ chemical shifts for prediction of backbone dihedral angles using TALOS+ [25]. The analysis program CCPN [40] was used to visualize NMR spectra.

NOESY crosspeaks were peak-picked and integrated manually, then the NOESY peaks were assigned and an ensemble of structures was calculated automatically using CYANA [27]; the tolerances used for assigning NOESY crosspeaks were 0.025 and 0.020 ppm in the F1 and F2 dimensions, respectively. ^1^Hα, ^13^Cα, ^13^Cβ, and ^15^N chemical shifts were used in TALOS+ to obtain predictions for the backbone φ and ψ dihedral angles; these were converted to dihedral-angle restraints for use in CYANA using an error range corresponding to twice the standard deviation estimated by TALOS+. Five hydrogen bonds were clearly identified in preliminary rounds of structure calculation, and the corresponding backbone amide protons were found to exchange slowly with solvent water based on a series of 2D TOCSY and 1D NMR spectra collected after dissolution of lyophilized peptide in D_2_O. Thus, hydrogen bond restraints of 1.7–2.2 Å and 2.7–3.2 Å were used for the H_N_-O and N-O distances, respectively, in subsequent rounds of structure calculations [28].

**Figure 7:**
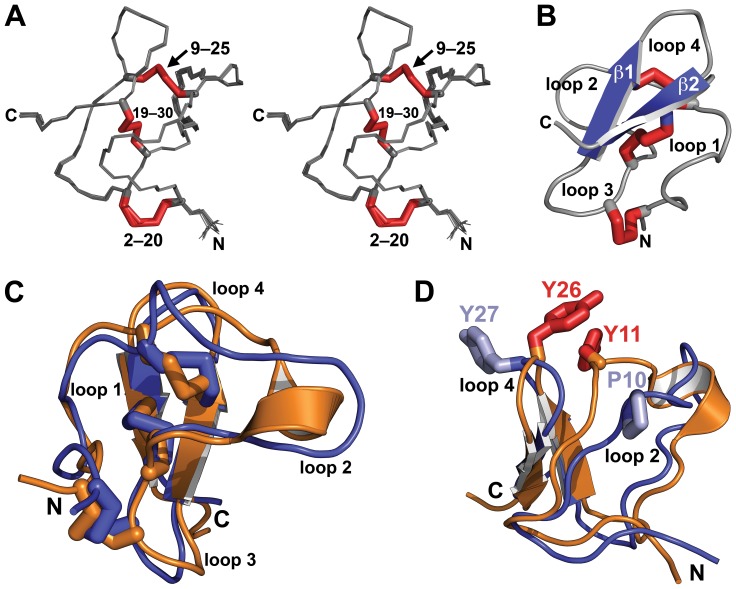
Structure of OAIP-1. (A) Stereoview of the ensemble of 20 OAIP-1 structures. The three disulfide bonds and the N- and C-termini are labeled. (B) Schematic (Richardson) representation of OAIP-1. β-strands are colored blue and disulfide bonds are shown as red tubes. The four intercystine loops (loops 1–4) are labeled. (C) Overlay of OAIP-1 (blue) and the orthologous toxin U_1_-TRTX-Pc1a (orange). The intercystine loops and termini are labeled. (D) Overlay of OAIP-1 (blue) and the orthologous toxin U_1_-TRTX-Pc1a (orange). Residues Y11 and Y26 in U_1_-TRTX-Pc1a (red tubes) interact in such a way that loops 2 and 4 are brought into close proximity. The equivalent residues in OAIP-1, P10 and Y27 (light blue tubes), do not interact and consequently loops 2 and 4 are further apart.

In the final round of structure calculations, 100 structures were calculated from random starting conformations, then the 20 conformers with the lowest CYANA target function values were used to represent the solution structure of sOAIP-1. CYANA assigned 87% (1098 out of 1262) of the NOESY crosspeaks during the automated structure calculations. The structural ensemble (Fig. 7A) has very high stereochemical quality, with very few steric clashes (as indicated by the low clashscore), no Ramachandran outliers, and a low percentage of unfavorable sidechain rotamers (Table 1). The highest-ranked member of the sOAIP-1 ensemble received a MolProbity score [41] of 1.69, placing it in the 89^th^ percentile relative to all other structures. Atomic coordinates for sOAIP-1 have been deposited in the Protein Data Bank (PDB) with accession number 2LL1.

**Table 1:** Structural statistics for the ensemble of OAIP-1 structures^1^.

Experimental restraints^2^	
Interproton distance restraints	
Intraresidue	135
Sequential	202
Medium range (i–j<5)	103
Long range (i–j≥5)	173
Hydrogen-bond restraints^3^	10
Disulfide-bond restraints	9
Dihedral-angle restraints (φ,Ψ, χ_1_)	49
Total number of restraints per residue	20.0
R.m.s. deviation from mean coordinate structure (Å)	
Backbone atoms (residues 1–33)	0.14±0.02
All heavy atoms (residues 1–33)	0.62±0.07
Stereochemical quality^4^	
Residues in most favored Ramachandran region (%)	93.4±0.7
Ramachandran outliers (%)	0±0
Unfavorable sidechain rotamers (%)	13.4±2.9
Clashscore, all atoms^5^	0.1±0.5
Overall MolProbity score	1.8±0.1

1 All statistics are given as mean ±S.D.

2 Only structurally relevant restraints, as defined by CYANA, are included.

3 Two restraints were used per hydrogen bond.

4 According to MolProbity (http://molprobity.biochem.duke.edu).

5 Defined as the number of steric overlaps >0.4 Å per thousand atoms.

Fig. 7B shows a ribbon representation of the ensemble of 20 sOAIP-1 structures. The structure comprises three disulfide bonds that form a classic inhibitor cystine knot (ICK) motif [42] in which the Cys2–20 and Cys9–25 disulfide bonds and the intervening sections of polypeptide backbone form a 14-residue ring that is bisected by the Cys19–Cys30 disulfide bond. A β-hairpin, which often houses the functionally important residues in ICK toxins [20], projects from the disulfide-rich core of the toxin; the two β-strands are formed by residues 23–26 and 29–32.

### Structural homologues of OAIP-1

The closest sequence match to OAIP-1 is U_1_-TRTX-Pc1a (62% identity), for which a 3D structure was previously determined [37]. The two structures overlay well with a backbone RMSD of 1.07 Å over 174 atoms (Fig. 7C). The major structural difference is an α-helix spanning residues 12–16 in U_1_-TRTX-Pc1a. An additional conformational difference is the presence of two tyrosine residues (Tyr11 and Tyr26) in U_1_-TRTX-Pc1a that interact and bring intercystine loops 2 and 4 close together (Fig. 7D). The corresponding residues in OAIP-1 (Pro10 and Tyr27) do not interact, and hence the corresponding backbone regions are well separated (Fig. 7D). However, the absence of this interaction does not appear to significantly change the overall conformation of the toxin. U_1_-TRTX-Pc1a was reported to have *in vitro* activity against the intra-erythrocyte stage of the malaria parasite *Plasmodium falciparum* [37] but its molecular target is not known. Thus, the sequence and structural homology with U_1_-TRTX-Pc1a unfortunately provides no insight into the likely molecular target of OAIP-1.

**Figure 8:**
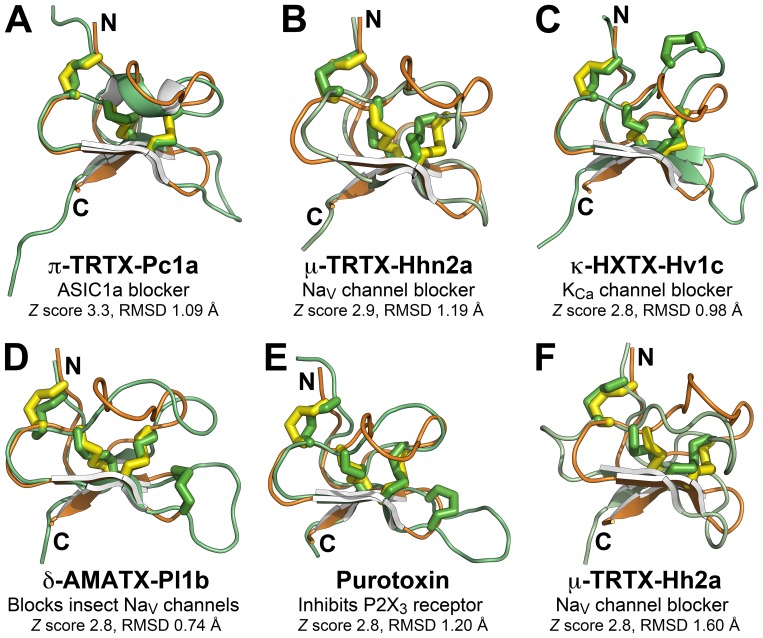
Structural homologues of OAIP-1. Alignment of the structure of OAIP-1 (orange) with the top six structural homologues (all shown in green) as ranked by the Dali server [43]. The activity of each structural homologue is indicated, as is the *Z* score and RMSD of the alignment. Disulfide bonds are shown as solid tubes and the N- and C-termini are labeled.

A broader search for structural homologues of OAIP-1 using the Dali server [43] produced 47 structural matches with a statistically significant *Z* score ≥2, almost all of which were toxins from spiders or venomous marine cone snails. However, the best six matches were all with ICK toxins from spider venoms; an alignment of sOAIP-1 with each of these toxins is shown in Fig. 8. Three of the six closest structural homologues of OAIP-1 block either insect or vertebrate voltage-gated sodium (Na_V_) channels. Superficially, this might appear to provide a clue as to the molecular target of OAIP-1. However, a block of Na_V_ channels would not induce the excitatory phenotype noted in insects following injection of OAIP-1, and hence this is unlikely to be its mechanism of action. The closest structural homolog of OAIP-1 according to Dali is π-TRTX-Pc1a (Fig. 8A), which is the most potent blocker known of acid-sensing ion channels (ASICs) [44], [45]. However, ASICs are restricted to chordates, so this channel cannot be the target of OAIP-1. Another structural homolog of OAIP-1, purotoxin (Fig. 8E), is a potent modifier of vertebrate P2X_3_ receptors, causing a concentration-dependent prolongation of channel desensitization [46]. However, as for ASICs, P2X_3_ receptors are not found in insects [47], so these receptors cannot be the invertebrate target of OAIP-1.

The only structural homologue that might provide some insight into the target of OAIP-1 is the insecticidal toxin κ-HXTX-Hv1c from the Australian funnel-web spider *Hadronyche versuta*. Like OAIP-1, this toxin induces an excitatory phenotype when injected into insects [48] or when the toxin is expressed in *Drosophila melanogaster* [49]. The target of κ-HXTX-Hv1c has proved enigmatic, but it is known to be a potent blocker of insect calcium-activated potassium (K_Ca_) channels [50]. OAIP-1 and κ-HXTX-Hv1c have low sequence identity (39%), but the two structures overlay closely with an RMSD of 0.98 Å (Fig. 8C). However, with one exception, the functionally important residues in κ-HXTX-Hv1c [51] are not conserved in OAIP-1. Thus, despite their similar 3D structures and the fact that they both induce an excitatory phenotype in insects, it is entirely conceivable that κ-HXTX-Hv1c and OAIP-1 have completely different modes of action. This is not entirely surprising since, as noted previously, the ICK scaffold is relatively insensitive to changes in intercystine residues [52], which has enabled spiders to develop diverse pharmacologies based on this single protein fold; as a result, structural homology between ICK toxins often provide little insight into toxin function [17].

## Discussion

### Functional activity of sOAIP-1

Development of a termiticidal assay enabled isolation of an orally active insecticidal peptide (OAIP-1) from the venom of *Selenotypus plumipes*, a large tarantula (theraphosid) native to arid-zone grassland regions of Australia. Although *S. plumipes* is one of Australia's largest spiders, with mature specimens having a legspan in excess of 16 cm, it is not harmful to humans. As far as we are aware, this is the first time an insecticidal venom peptide has been isolated using an assay based on oral activity.

Synthetic OAIP-1 was successfully produced via chemical synthesis. Exposure to acetonitrile, TFA, repeated lyophilization, low pH, or temperatures up to 30°C for one week did not affect the activity or RP-HPLC profile of the toxin, indicating that it is highly stable. Thus, chemical synthesis is a plausible route for large-scale production. The activity of synthetic OAIP-1 was confirmed *in vivo* against several taxonomically divergent insect pests, including cotton bollworms, mealworms, and termites, but there were significant phlyum-specific differences in activity. In particular, the oral insecticidal activity of the toxin against cotton bollworms, a pernicious pest of cotton and other crops, was 1644-fold higher than against mealworms, a stored grain pest (LD_50_ values of 104 pmol/g and 171 nmol/g, respectively). Although we did not measure the LD_50_ for oral administration of OAIP-1 to termites, it is expected to be similar to that for mealworms given that a single dose of 350 nmol/g caused ∼70% mortality (Fig. 1C).

For nuisance pests like cockroaches, filth flies, and blood-feeding mosquitoes, a repellent insecticide can be useful. However, in the case of insecticides used to target pest social insects (termites, ants, wasps), a non-repellent insecticide that can be horizontally transferred is more desirable. Based on the results of the choice test, it appears that OAIP-1 is not repellent. Experiments using lipophilic tracking dyes could be used to determine whether the insecticide could be horizontally transferred, an important feature for commercially-viable termiticides [53].

### Structural characterization of sOAIP-1

OAIP-1 is a member of a large class of disulfide-constrained peptides known as knottins [54]. Despite being structurally similar to several other spider toxins, these structural homologies provide few clues about the molecular target of OAIP-1. Perhaps the most intriguing structural homology is with another excitatory insecticidal neurotoxin, κ-HXTX-Hv1c. However, the pharmacophore residues previously elucidated for κ-HXTX-Hv1c [51] are not conserved in OAIP-1 and there are significant differences between the two structures despite the overall similarity in their backbone conformations. For example, the five-residue β-hairpin loop that houses several of the pharmacophore residues in κ-HXTX-Hv1c is much shorter in OAIP-1, comprising only two residues (Fig. 8C).

The other structurally homologous arachnid toxins have activity in vertebrates, but their insecticidal activity has often not been considered or determined. Of the non-arachnid structural homologs, several less close matches provide an interesting context for the potential evolution of sOAIP-1 as a venom component. VHv1.1 from the venom of the parasitic wasp *Campoletis sonorensis* (Hymenoptera: Ichneumonidae) was the ninth closest structural homolog of sOAIP-1 [55]. This toxin induces sublethal effects when fed to pestiferous larvae of the noctuid moths *Heliothis virescens* and *Spodoptera exigua* [56]. Matches 20, 22, and 30 were to antimicrobial tachystatins isolated from horseshoe crab hemolymph, a component of the crustacean's immune response [57]. Thus, OAIP-1 might have been recruited into spider venom by duplication of an ancestral tachystatin-like gene. Interestingly, tachystatins bind chitin, which, in addition to being the major component of the insect exoskeleton, is also found in the peritrophic matrix, a sac-like structure that surrounds the gut lumen of most insects. It should be interesting to examine whether chitin binding plays a role in the ability of OAIP-1 to traverse the insect gut epithelium in order to reach its presumed nervous system target.

### Application of OAIP-1 to insect pest control

Table 2 compares the oral toxicity of OAIP-1 against *H. armigera* with that of several commercially available pyrethroid insecticides. Remarkably, on a molar basis, OAIP-1 is more potent than any of these chemical insecticides. The oral potency of OAIP-1, its rapid insecticidal action (i.e., death within 24–48 h), and its facile production via chemical or recombinant methods makes this peptide a good candidate for deployment as a foliar spray against lepidopterans and possibly other pest insect species. Moreover, the toxin should degrade in the environment to innocuous breakdown products.

**Table 2:** Comparison of OAIP-1 with pyrethroid insecticides.

Insecticide	Class of insecticide	Strain	Oral LD_50_ (nmol/g)	Reference
Bifenthrin	Pyrethroid	R^1^	20.6	[61]
		S^2^	1.1	
Deltamethrin	Pyrethroid	R	0.46	[61]
		S	0.35	
Etofenprox	Pyrethroid	R	55.9	[61]
		S	0.31	
Fenvalerate	Pyrethroid	R	41.9	[61]
		S	7.8	
OAIP-1	Peptide	–	0.10	Current study

1 Pyrethroid-resistant strain BK99R9.

2 Susceptible strain BK77.

Since OAIP-1 is a genetically encoded peptide toxin it should also be possible to engineer transgenes encoding OAIP-1 into plants. Transgenes encoding insecticidal spider-venom peptides have already been used as an insect-resistance trait in cotton [58], poplar [9], and tobacco [8], [59]. While the introduction of crops expressing insecticidal δ-endotoxins (also known as Cry toxins or simply *Bt*) from the bacterium *B. thuringiensis* has revolutionized global crop production, there are concerns that constitutive expression of *Bt* in transgenic plants will ultimately expedite resistance development. An OAIP-1 transgene might be good candidate for trait stacking with *Bt* since: (i) it has a completely different mechanism of action; (ii) OAIP-1 activity is likely to be synergized by *Bt*, which causes lysis of midgut epithelial cells [11] and therefore should facilitate OAIP-1 movement into the hemocoel; (iii) whereas *Bt* toxins are specific for the insect orders Lepidoptera, Coleoptera, Hymenoptera and Diptera, OAIP-1 is likely to have a broader range of insecticidal activity.

An OAIP-1 transgene could also be used to enhance the efficacy of insect pathogens. A wide range of bacterial, viral, protozoan, and fungal pathogens can infect insects. Many of these have potential as bioinsecticides, and some fungal entomopathogens are already used commercially [60]. However, a major disadvantage of many of these entomopathogens is their slow kill time (typically >7 days). It was recently demonstrated [10] that the potency and speed of kill of the entomopathogenic fungus *Metarhizium anisopliae* against mosquitoes and locusts could be substantially improved by engineering it to express AaIT, an insecticidal peptide derived from scorpion venom. Engineering this fungus to express OAIP-1 might confer a similar enhancement of potency and kill time.

In summary, we have isolated an orally active insecticidal peptide from spider venom that is more potent against cotton bollworms, an extremely important agricultural pest, than many chemical insecticides. This peptide could potentially be deployed as a foliar spray or a transgene encoding the peptide could be used as an insect resistance trait in crop plants or be used to enhance the efficacy of insect pathogens.

## Supporting Information

Table S1 **Components of the agar-based insect diet used for oral toxicity studies.**
(DOCX)

Table S2 **Scoring scheme for phenotypic response of mealworms to injection of sOAIP-1.**
(DOCX)
