# Supplementary material for: Isolation of an Orally Active Insecticidal Toxin from the Venom of an Australian Tarantula
Source: PLoS One. 2013 Sep 11;8(9):e73136. doi: 10.1371/journal.pone.0073136 (PMC3770646; doi:10.1371/journal.pone.0073136)
Supplement: Table S1 — Components of the agar-based insect diet used for oral toxicity studies. (DOCX) [file pone.0073136.s001.docx]

**Table S1**: **Components of the agar-based insect diet used for oral toxicity studies**

| **Components** | **Amount in 50 mL** | **Supplier** |
| --- | --- | --- |
| Agar | 0.56 g | Fischer Biotech, Fairlawn, NJ |
| Tryptone | 4.1 g | Bacto-Tryptone, BD Sparks, MD |
| Yeast | 2.4 g | Sigma-Aldrich, St Louis, MO |
| Sucrose | 10 g | Merck, Darmstadt, Germany |
| Pentavite | 90 μL | Bayer, Leverkusen, Germany |
