# Supplementary material for: Isolation of an Orally Active Insecticidal Toxin from the Venom of an Australian Tarantula
Source: PLoS One. 2013 Sep 11;8(9):e73136. doi: 10.1371/journal.pone.0073136 (PMC3770646; doi:10.1371/journal.pone.0073136)
Supplement: Table S2 — Scoring scheme for phenotypic response of mealworms to injection of sOAIP-1. (DOCX) [file pone.0073136.s002.docx]

**Table S2**: **Scoring scheme for phenotypic response of mealworms to injection of sOAIP-1**

| **Response** | **Score** | **Characteristics of phenotypic response^1^** |
| --- | --- | --- |
| Excitatory | 2 | Movements more erratic than control insects |
| Excitatory paralysis | 1 | Movements so erratic that the insect is effectively paralyzed and not able to move independently |
| Dead or moribund | 0 | Insect is dead or unable to right itself when turned  on its back |
| Depressive Paralysis | –1 | Movements so depressed that the insect is effectively paralyzed and not able to move independently |
| Depressive | –2 | Movements depressed compared with control insects |

^1^Insects were assessed at 5, 30, and 60 min post-injection. The observed states were given a numeric score based on differences from control insects injected only with water in order to determine whether the peptide was excitatory or depressive.
